# Supplementary material for: We choose: Adolescent girls and young women’s choice for an HIV prevention product in a cross-over randomized clinical trial conducted in South Africa, Uganda, and Zimbabwe
Source: PLoS One. 2024 Aug 29;19(8):e0308577. doi: 10.1371/journal.pone.0308577 (PMC11361692; doi:10.1371/journal.pone.0308577)
Supplement: Attachment — Submitted filename: Response to reviewers_PONE_D_23_24059_16May2024.pdf [file pone.0308577.s002.pdf]

May 16, 2024

Dear reviewers

Thank you for your additional review of this paper. We appreciate your attention to this paper and your valuable comments. Our response to your comments is outlined below:

4: Data availability: We have noted the PLOS data policy which requires authors to make all data underlying the findings described in their manuscript fully available without restriction, with rare exception (please refer to the Data Availability Statement in the manuscript PDF file). Due to the composition of the study sites with varying confidentiality agreements, our response is as follows: Study data are available upon request from the Microbicide Trials Network by submission of a "Dataset Request Form" available at http://www.mtnstopshiv.org/resources. Interested parties would be able to access these data in the same manner as the authors. The authors did not have any special access privileges that others would not have."

Our response to the specific reviewers' comments is outlined below:

Reviewer #1: Dear authors, the arguments shown in the rebuttal and the modifications that you propose are satisfactory. However, with regards to point 9. You wrote in the rebuttal that you added in the manuscript text "To make it clearer, we have stated that 'research examining product preference and choice using products with active HIV prevention drugs is still lacking, and this is the first of its kind testing preference for oral PrEP containing Emtricitabine/Tenofovir Disoproxil Fumarate and the vaginal ring containing Dapivrine, by offering participants the option to experience both products before choosing their preferred HIV prevention product' However, this info was not shown in the revised version of the manuscript. Please revised the manuscript and ensure that it corresponds with what is mentioned in the rebuttal. It is not feasible for me to check whether the revised manuscript corresponds with what you mention in the rebuttal as modification.

RESPONSE: We apologise for the oversight and thank you for bringing this to our attention. We have included the statement…*'research examining product preference and choice using products with active HIV prevention drugs is still lacking, and this is the first of its kind testing preference for oral PrEP containing Emtricitabine/Tenofovir Disoproxil Fumarate and the vaginal ring containing Dapivrine, by offering participants the option to experience both products before choosing their preferred HIV prevention product',* on page 3 of the paper.

Non-Profit Company : Registration no. 1999/005072/08 : NPO no. 148-956
Public Benefit no. 18/11/13/51
Directors: Prof L-G Bekker; Ms Z Ebrahim (Chair); Ms T Tutu-Gxashe; Mr P Grant; Ms M K Ndebele; Dr A M Kubeka; Mr K Osborne; Mr R Appelbaum
www.desmondtutuhealthfoundation.org.za

Reviewer #1 comment: Point 10 in your rebuttal: "What determined the number of FGD and IDI? Was any interim analysis conducted?". You refer to point 4. Thank you for the info. You refer to information power. How did you assess whether you had collected enough information, and that additional data collection would not result in additional new insights (thus whether saturation was reached)?

RESPONSE: Correct, we conducted interim analysis using what we call debriefing reports. We thus have added a statement that…'*Additionally, during data collection, we promptly analysed debriefing reports (DRs) from completed interviews before the conclusion of data collection. These debriefing reports synthesized the main themes emerging from each interview, with interviewers completing them within 4-7 days of conducting the interview. This rapid analysis enabled us to ascertain data saturation.* This is added on page 33.
